# Dr. Wood, on Vaccination

**Published:** 1805-01-01

**Authors:** J. Wood

**Affiliations:** Newcastle upon Tyne


### *To the Editors of the Medical and Phyſical Journal.*

GENTLEMEN,

I Have very often proposed to myself to address you; but sometimes the difficulty of the first introduction has prevented me; sometimes the subject on which I meant to write did not, on a second view, appear sufficiently important or new for a first address; sometimes I waited until the following month, when I found my observations anticipated, and very ably too, by a Candidus or a Medicus; and sometimes I deferred, through indolence I fear, giving you certain facts or remarks until the period was past from which they would derive if not great part of their value, at least the salt to render them interesting Neither of these can I fear on the subject of vaccination, for its value must be ever independent of times and seasons; and it wants not any salt to render it interesting: its value, like gold, will be stable and certain; and though it may sometimes be adulterated with too much alloy, the fire will again render it pure; and such is the trial vaccination is now undergoing. Perhaps I am wrong in thus giving you my sentiments, before I have stated the experience from which they have sprung; but as the importance of the subject to mankind is the stimulus which has induced me to bring forward my testimony, so I may be excused allowing some of its operation in this preface. I am deeply sensible too, and I wish that medical men in general were more so, of the fault of omission, as well as commission, or, in other words, that I may be negatively as well as positively unjust. I say, I wish that medical men were in general more impressed with the idea that it is possible to be guilty of the first species of injustice; for I am sure, if they were, many valuable facts would not be lost to the world. It may, and I dare say has been said by individuals, as well on the subject of vaccination as of influenza and others, "I need not give my opinion, nor my testimony, there are a sufficient number to do so without me; mine will be lost, or be superfluous, in the general voice." But they must recollect, that many particles constitute a heap of sand; and they do not consider, that if all were to reason in like manner, all would be silent. I have made this latter remark, as I feel it applicable to myself at present; I mean, though the testimony I were to give you may be considered as small particles, yet I think it right to add them to the general heap. When

When the cow-pox was first named here, I believe it was only *with a smile*, as in many other places; yet I do not think we were here any later in adopting it, than in other parts of the kingdom; for the practice commenced in this place in the end of 1799, and was very general in 1800. In May, 1801, now three years and a half ago, my then youngest child was inoculated with the cow-pox matter, and the arm exhibited the usual signs of the genuine disease, which was communicated to the system in an evident manner. The year following I had another child inoculated with the cow-pox matter, (now two years and a half ago) and it went through the disease in as satisfactory a manner as the first. I then determined to inoculate them both with variolous matter; but by this time variola had totally disappeared in this neighbourhood, as it was not to be found within an extensive circuit. Sometimes I was told of its being in a neighbouring village; but on farther inquiry, or inspection, it proved to be the chicken-pox, or some similar eruption.* The small-pox being thus, as it were, extinguished, I was more indifferent about the second inoculation; still my inquiries, with those of Mr. Ingham, surgeon, who inoculated my children, continued; and it was only within these few weeks that Mr. Ingham succeeded in procuring some matter. It is supposed the small-pox was at last brought to this neighbourhood by some of the military, or by the cloaths of some of their children. My two young folks were therefore inoculated with the variolous matter on the 14th of last month. The punctures began to inflame within six hours after the insertion of the matter; the inflammation was very considerable on the following day, and increased to the fourth day, when it began to subside; on the fifth day a second inflammation began, with appearance of suppuration about the wounds, which went on till the seventh, when it began again to subside; and it nearly disappeared on the 10th, leaving an irregular scab on the surface of the suppuration. The children kept perfectly well, not being in the smallest degree affected with the presence of a *large quantity of variolous matter* in their arms. I could not perceive even the axillary glands affected in either.

This

* The chicken-pox is called here by different names, vulgarly, the *water-jags*; a little farther north, I believe, it is sometimes called the *horn-pox*; and a little farther, the *swine-pox*. Whether, however, these different names are applied to the same species of eruption, I do not assert positively; and will be glad to be informed by any of your Correspondents.

This was the experiment decisive of the security of the vaccine disease against the small-pox, at the distance of three years and six months in the one case, and of two years and six months in the other. I ought to mention, that the youngest, at the time of being vaccinated, was very much affected with the eruption peculiar to young children, well known by the name of the *red gum*, or the *strophulus* of Dr. Willan, which was attended with convulsion, and was wholly relieved from this troublesome complaint by the vaccine disease, and has continued ever since in perfect health. The child had, before vaccination, at various times, different species of this eruption. When I first wished to inoculate my children with small-pox after the vaccine disease, I entertained, I confess, *some small* degree of scepticism, which is, in my opinion, not to be condemned in medical men. That unbelief had been long ago removed; and I now inoculated them with small-pox matter, as a duty I owed them and the public *at this particular period*. This duty has been also felt by several gentlemen of the profession in this place, who have inoculated children for the small-pox after vaccination many years ago, with the same result as I have above stated. It was my intention to have waited for the *exact dates* of some of those cases, as, in four at least, more than four years elapsed between the two inoculations; but as *they* have not yet reached me, I will not now wait for them.

Just as I had written the above, I received a report of two of the cases alluded to, from Mr. Horn, surgeon, in this town, which I will send you as it is; and if it should appear to you too long for insertion, you can give the substance of it, as Mr. Horn requests.

To JAMES WOOD, M. D.

Dear Sir,

I MUST make my situation since I saw you on Thursday last, (having never left my room, where I have been fast moored by my inveterate enemy the gout) plead my excuse for not having complied sooner with your request.

Master C. a fine healthy boy, near four years of age, who had passed through the vaccine disease in his early infancy, was, at the request of his friends, inoculated by me on the 2d of November, 1804, with fresh fluid *variolous matter*, taken that morning. I made a slight puncture in one arm, avoiding as much as I could to injure the cutis; and then re-inserting the point of my lancet, well well charged with fluid matter, (taken from a piece of lint which had been soaked in several well maturated pocks on the 7th day from the eruption) I endeavoured, by pressing down the cuticle with my nail while the lancet was underneath the skin, to leave the matter between the cutis and the cuticle; and in doing this, the least possible *speck* of blood appeared. On the other arm I made the slightest scratch, for about one-sixth of an inch in length, through the cuticle, so as just to reach the cutis, and then separating, or rather stretching, the two sides of the little scratch, I wiped the flat part of the lancet, well charged, upon the skin. Next morning, the 2d of November, both the puncture and scratch were much inflamed, and the redness extended to the circumference of a half-penny; the 4th of November, and third day from the operation, reckoning the day it was performed as the first, the redness was much less, and I was a little in doubt; the 6th of November the redness was entirely gone off, and a little scabby appearance remained, which disappeared in two days, and the child never felt any other symptom whatever. I cannot give you the exact age of my patient, he having been taken home to London; neither can I tell you the exact distance of time from the *vaccine inoculation*. I am, however, pretty certain that he was near four years old; and I am sure he was vaccinated very early, under a practitioner of high celebrity in town.

On the 3d of November, 1804, I inoculated Miss B. between four and five years of age, and who had been vaccinated above four years before by your late friend, Keenland, when she shewed every appearance of the genuine disease. The variolous matter was taken from the same patient from whom I procured that employed for Master C. The same method was used, and the same appearances followed, with rather less redness on the second day; but yet with far more than usually appears in small-pox inoculation at the same period.

These are the only two I have inoculated with variolous matter since the doubts respecting the preventive effects of vaccination have been made public. I have however inoculated several, I believe I may say scores of children, who had recently been vaccinated, and no particular effect followed.

I have lately, i.e. the 29th of October last, vaccinated a young lady, of about nineteen or twenty years of age, who had, at different periods of her life, been three times inoculated with variolous matter; but no disease ever followed, lowed, as far as I could learn, although three distinct pits or marks from the different inoculations were very visible. I took the vaccine matter from a well-defined pock on the arm of a healthy child the eighth day from the time it was vaccinated (still reckoning the day of the operation as the first.) The appearances on her arms were such on the second and third days, as to induce me to suppose the disease would follow. There was a slight redness and rising on each of the parts punctured and scratched, and she complained of stiffness and slight pain up the arm, extending to the axilla; on the fourth day these appearances declined, and on the seventh there were none remaining.

I am, &c.

*Newgate Street, Dec. 5,* 1804. FREDERICK HORN.

Next to the vaccine disease being a security against the small-pox, I consider the circumstance now fully established, that the second inoculation with vaccine matter, if the first has affected the system, does not produce a pustule similar to the first, or, in other words, which goes thro' the stages of the original vaccine pustule; of next importance, for if there is any doubt of the security produced by the first inoculation, a second inoculation only is necessary to remove all doubt, as if the pustule does not come forward in the regular way, but retains something similar to the small-pox after vaccination, a perfect security may be relied on, without having recourse to small-pox inoculation, which only keeps alive that dreadful disease, and tends to prevent its total extinction. On this point it may be curious to observe, that although the first vaccine pustule produced by the usual inoculation does not sometimes exhibit all the signs of a genuine pustule, by inflammation taking place in a great degree, yet the system is equally secure against the small-pox; and on a second inoculation with vaccine matter, the pustule does not go forward as soon as if the order of the first pustule had not been injured by too great inflammation.

It is yet the custom here with the common people to bring their children for inoculation to the Dispensary in the spring and autumn; it is therefore necessary either to procure fresh matter for each of these periods, or preserve some in the best manner possible. Some vaccine matter was preserved last winter, nearly for six months, at the dispensary here, in capillary glass tubes hermetically sealed; it had been preserved so before, and answered very well. This last spring, all the children inoculated with this matter matter had their arms violently inflamed,* and instead of the regular pustule and scab, a large crust of a brown colour with ulceration took place. Much apprehension for the security of so many children, about two hundred and fifty, naturally took place, and the inoculation was stopped, until fresh vaccine matter was procured from the Vaccine Institution in London. On procuring this, the inoculation went forward, and the usual pustule was produced. Altho' some of the arms of the first inoculation did not go on to the degree mentioned above, yet matter taken from them by private practitioners in the town, produced equal inflammation on the second remove; notwithstanding the violence of the inflammation, the security wished for was obtained without a general re-inoculation, (which would have been impossible) by all those who were inoculated a second time with the fresh vaccine matter, *resisting its second action,* in the manner I have mentioned before; and out of the whole number,† there has not occurred an instance of any one being seized with the small-pox, altho' it has made as much progress here of late as it seemed to be capable of. To give a general idea of the state of the arms of these children at the Dispensary, whose arms were so much inflamed as to destroy the progress of the regular pustule, and also of the difference of the pustule in the second inoculation, from the first, I will give one case as stated to me by Mr. Fife, surgeon, which occurred in his practice.

" That

---

* The inflammation was so violent in two or three instances as to be supposed to produce an eruptive vaccine disease. In two cases I saw an eruption; the one appeared to me, and many others who saw it, to be *accidental* chicken-pox; the other was merely *a rash,* of an erysipelatous kind, not sensible to the fingers.

† The vaccine inoculation began by the Dispensary here, in March 1801; and the following is the report to the 3d of December last, from that period.

| Inoculated in | | Had the disease. | | Did not take the disease. |
|---|---|---|---|---|
| 1801, 2, 3, - | 921 | 917 | — | 4 |
| To Sept. 1804 - | 451 | 451 | — | 0 |
| To Dec. 3, 1804 | 186 | 186 | — | 0 |
| | ——637 | ——— | | — |
| | —— | 1554 | | 4 |

Total - 1558 inoculated; and not one instance has occurred of any having been afterwards affected with small-pox, or any eruption like it. In the former reports of the inoculation of the small-pox, the number that did not take the disease, formed every year a much larger proportion than those that did not take the vaccine disease. The above report shews how much vaccination increases in favor here with the common people, who were invincibly averse to inoculation for the small-pox.

"That part of the arm where the matter was inserted, put on the usual appearance till about the fifth day; it then looked as though the vesicle had burst: the inflammation from this time increased considerably, and instead of a distinct pustule, a large irregular crust formed, of nearly the size of half-a-crown, and successive scabs continued forming for near a month."

A second vaccination was attempted in this case two years and a half after, and the regular pustule did not take place; but after a hasty inflammation, which continued several days, at an equal degree, a small vesicle and scab were formed, without any areola, or succeeding scab, as takes place in the regular pustule on the first inoculation, when too much inflammation* does not prevent it.

The next circumstance deserving attention is, that in a second inoculation with small-pox matter, after vaccination, the appearance of the scab is different from the scab that follows the suppuration of the inoculated part in a first inoculation with small-pox matter previous to vaccination; this was first observed to me by Mr. Fife, who describes the difference as follows:

"When a child has been inoculated with small-pox, (not having before been vaccinated) the succeeding scab is generally of an irregular form, the colour resembling horn, and the edge very little elevated above the surface of the skin. In the trial I made with my own child with variolous matter after vaccination, an irregular crust was formed, which died away after the seventh day."

On the arms of my own two children, mentioned before, the same irregular crust was formed, and different from that which follows inoculation with small-pox matter *before* vaccination.

This observation appears to be of *much importance*, as it will tend to prove in all second inoculations with small-pox matter after supposed vaccination, whether the system had

* It has been observed, that any external sore, or inflammation, is evidently affected when the system is under the influence of the vaccine disease; in the case mentioned by Mr. Fife, he says, "The child, when first vaccinated, had a slight eruption nearly surrounding the right ear; after the irregular crust was formed, the eruption below the ear became much worse, degenerating into several small foul ulcers, which continued open near a month; the inoculated part continued forming successive scabs during the same time, and both got well nearly together." Was not the affection of the inflamed part near the ear, in this case, an evident sign of the system being under the influence of the vaccine disease? The second inoculation certainly went to prove this.

had been really vaccinated or not; as, if it had, no eruption of course would take place, and the irregular scab would, or, vice versa, if eruption of small-pox took place, the appearance of the scab might, certify that the system had not been previously vaccinated.

My wish to send you this report in time for your next number, prevents my having the opportunity of rendering it so compleat as I at first proposed;* but I am induced to send it as it is, as you have not had any report whatever of vaccination from this district, which, considering its extent, and its population, is the more surprising. At the last meeting of the Medical Society of this town, the subject of vaccination, with the late cases of Mr. Goldson and Dr. Rollo, was fully discussed, and the Society agreed to the following conclusion: *That from our experience, we have no doubt whatever of the vaccine disease being a security against the small-pox, but only from what we have read.*

To this conclusion I will add a few more, drawn from what I have seen.

That the vaccine disease is a security against the small-pox, even although the pustule has been prevented, by too much inflammation, from assuming the regular appearance described by it.

That the eruption peculiar to infants, called *strophulus*, does not prevent vaccination being a security against the small-pox, although present when the inoculation takes place.

That after an effectual inoculation with vaccine matter, a second inoculation with the same matter does not produce similar effects, by the pustule not going on as in a first inoculation.

That the scab formed on the suppurated part produced by inoculation with the small-pox matter, differs in that performed after vaccination from that which takes place on inoculation with small-pox matter before vaccination.

Thus we are not infected here with any improper scepticism or infidelity. We know that there are neither general rules without some exceptions, nor general laws without some varieties; and we believe that the vaccine disease will have as few anomalous cases as the dreadful disease had, which it has, I trust, exploded; and that should it *ever happen* that small-pox takes place after satisfactory vaccination,

* I intended to have made my inquiries general by a circular letter to all the surgeons in this neighbourhood; but I found the undertaking would be too great for my time *at present*.

vaccination, we are no worse than when small-pox took place after small-pox, but much better, because the chance of ever receiving the infection of small-pox will be so much diminished.

*Newcastle upon Tyne,*
*Dec.* 5, 1804.

I am, &c.
J. WOOD, M.D.

